# Optimization of Ultrasound-Assisted Extraction of Phenolic Compounds from Romanian Blackthorn (*Prunus spinosa* L.) Fruits

**DOI:** 10.3390/antiox14060680

**Published:** 2025-06-03

**Authors:** Ana-Maria Drăghici-Popa, Oana Cristina Pârvulescu, Raluca Stan, Ana-Maria Brezoiu

**Affiliations:** 1 Department of Organic Chemistry, National University of Science and Technology POLITEHNICA Bucharest, 1-7 Gheorghe Polizu St., 011061 Bucharest, Romania; ana_maria.draghici@upb.ro (A.-M.D.-P.); raluca.stan@upb.ro (R.S.); 2 Department of Chemical and Biochemical Engineering, National University of Science and Technology POLITEHNICA Bucharest, 1-7 Gheorghe Polizu St., 011061 Bucharest, Romania; ana_maria.brezoiu@upb.ro

**Keywords:** antioxidants, bioactive compounds, blackthorn, central composite design, Plackett–Burman design, phenolic compounds, *Prunus spinosa* L., ultrasound-assisted extraction

## Abstract

Selecting factors that significantly affect the extraction process and optimizing them are essential to obtain high extraction efficiency. This study aimed at optimizing the ultrasonic-assisted extraction (UAE) of polyphenols from Romanian blackthorn fruits using aqueous solutions of ethanol as green extraction solvents. Six process factors, including solvent/plant material ratio (*R_LS_* = 4.95–15.1 cm^3^/g), ethanol concentration in the extraction solvent (*c_et_* = 16.4–83.6%), extraction temperature (*t* = 30–70 °C), pH of the solvent (*pH* = 2–7), amplitude of the ultrasonic probe (*A* = 30–70%), and extraction time (*τ* = 5–15 min), were screened and optimized based on a Plackett–Burman design (PBD) and a central composite design (CCD). Statistical analysis indicated that *R_LS_*, *c_et_*, and *t* significantly affected the process response variables in terms of total phenolic content (*TPC*), total anthocyanin content (*TAC*), and antioxidant capacity (*AC*). Under optimal conditions (*R_LS_*_,*opt*_ = 15.1 cm^3^/g, *c_et_*_,*opt*_ = 33.2%, *t_opt_* = 66.8 °C, *pH_opt_* = 7, *A_opt_* = 50%, and *τ_opt_* = 10 min), the following levels of response variables were experimentally determined: *TPC_opt_* = 14.45 ± 0.718 mg GAE/g DM, *TAC_opt_* = 0.405 ± 0.057 mg C3GE/g DM, and *AC_opt_* = 16.75 ± 1.144 mg TE/g DM. Six phenolic compounds were identified in the extract obtained at optimal levels of process factors, i.e., rutin (7.12 ± 0.06 mg/100 g DM), protocatechuic acid (6.83 ± 0.01 mg/100 g DM), neochlorogenic acid (4.88 ± 0.01 mg/100 g DM), vanillic acid (3.70 ± 0.01 mg/100 g DM), chlorogenic acid (1.93 ± 0.02 mg/100 g DM), and caffeic acid (1.51 ± 0.01 mg/100 g DM).

## 1. Introduction

In recent years, researchers and food/pharmaceutical/cosmetic producers have paid more attention to underutilized wild plants, including *Prunus spinosa* L. (blackthorn) [1,2,3]. Various parts of this plant have been used for diet and medicinal purposes, especially its fruits, which have diuretic, purgative, spasmolytic, and astringent properties [1,3,4,5,6,7,8,9,10,11,12,13]. Blackthorn fruits, which are consumed either raw or processed (usually in the form of jam, jelly, marmalade, compote, juice, tincture, liqueur, and brandy), are used to treat various conditions/diseases, e.g., gastrointestinal, cardiovascular, respiratory, and urinary tract disorders, diabetes, obesity, inflammatory processes in the mouth and throat [3,9,13,14,15,16,17,18].

Besides significant amounts of phenolic compounds, such as phenolic acids and flavonoids (e.g., anthocyanins, flavonols, flavanols, flavanones, flavones, tannins), these fruits are valuable sources of carbohydrates, dietary fibers, monounsaturated fatty acids, essential minerals (including K, Ca, Mg, Na, Fe, Cu, Mn, Zn, and Ni), and vitamins C and E [3,8,14,15,16,19,20,21]. The chemical composition and health-promoting properties of blackthorn fruits can vary significantly depending on their genotype, geographical origin (altitude, sun exposure, temperature, precipitation, soil characteristics), and harvest time (usually from September to November) [16]. Numerous studies have highlighted antimicrobial, antioxidant, antidiabetic, anti-inflammatory, and anticancer effects of blackthorn fruit extracts [1,3,5,9,12,13,14,15,16,20,21,22,23]. These beneficial effects are mainly attributed to phenolic compounds, especially phenolic acids, flavonols, and flavones [16].

The extraction of phenolic compounds from blackthorn fruits can be performed by conventional methods, including maceration, percolation, hydrodistillation, Soxhlet extraction, or modern methods, e.g., ultrasound-assisted extraction (UAE) or microwave-assisted extraction (MAE) [2,24,25]. On the one hand, conventional methods usually involve long extraction times, large volumes of solvent (sometimes toxic), and higher temperatures, resulting in degradation of thermosensitive bioactive compounds, low extraction efficiency, environmental concerns, and high energy consumption and costs [26,27,28,29,30,31,32]. On the other hand, the use of ultrasound or microwaves can significantly improve the performance of the extraction process [20,24,26,27,30,32,33,34].

UAE is an eco-friendly technique that is widely used to extract bioactive compounds from plants due to its advantages, including shorter extraction time, higher extraction efficiency, lower solvent volume, energy consumption, and costs [24,30,33,34,35]. These positive effects are due to the acoustic cavitation phenomenon, which generally refers to the formation, growth, and implosion of cavitation bubbles formed from gas/vapour dissolved in the liquid. The implosion of these gas/vapour bubbles on the surface of plant material leads to micro-jets and shockwaves that act on plant structure through different mechanisms, e.g., fragmentation, erosion, ultrasonic capillary effect, sonoporation, and detexturation (destruction), whereas their implosion in the liquid phase results in macro-turbulences and micro-mixing [24,30,36]. All these effects determine an improvement in the mass transfer of solute species [24,27].

The most important parameters influencing the performance of the UAE process are characteristics of ultrasonic equipment (shape and size of ultrasound bath/probe, ultrasound power, intensity, frequency, and duty cycle), type, composition, and pH of extraction solvent, genotype, origin, harvest time, and pretreatment (e.g., chopping, drying) of plant material, and operation conditions, including liquid (extraction solvent)/solid (plant material) ratio, temperature, time, and number of extraction steps [1,16,24,30,37]. UAE is performed in bath or probe-type ultrasonic equipment at low ultrasound frequency (20–100 kHz) and high intensity (10–1000 W/cm^2^) [24,30]. An extraction time of 5–15 min is recommended [1,30]. Selecting factors (independent variables) that significantly influence the extraction process and optimizing these factors are essential to achieve high extraction efficiency [2,30,38].

In our previous work [39], the optimal conditions for the classical extraction (maceration under stirring) of phenolic compounds from Romanian blackthorn fruits (harvested in October 2019) using ethanol and its aqueous solutions as green extraction solvents were established. The effects of three quantitative factors, i.e., ethanol concentration in the extraction solvent, process temperature, and time, on the extraction performance were evaluated using the one-factor-at-a-time (OFAT) method. This method does not take into account factor interactions and does not always lead to finding optimal factor settings [2,40]. Moreover, the OFAT approach is not effective for a large number of factors [2,40]. To save time and reduce costs, Plackett–Burman design (PBD) can be applied to screen multiple independent variables and determine the most important ones, then response surface experimental designs, including central composite design (CCD) or Box–Behnken design (BBD), can be used to quantify the effects of these significant factors and their interactions on the extraction performance and to determine the optimal operating conditions [1,2,40,41,42,43].

In this study, the UAE process of phenolic compounds from Romanian blackthorn fruits (harvested in October 2023) using aqueous solutions of ethanol as extraction solvents was optimized based on a PBD combined with a CCD. Six quantitative factors (solvent/plant material ratio, ethanol concentration, pH of the solvent, amplitude of the ultrasonic probe, extraction temperature and time) were screened using a PBD to assess which factors significantly influenced the process performance. The optimal levels of these relevant independent variables, i.e., solvent/plant material ratio, ethanol concentration, and extraction temperature, were then found by applying a CCD.

## 2. Materials and Methods

### 2.1. Plant Material

Wild blackthorn fruits were harvested in October 2023 from Bănești (44°16′26″ N 25°53′27″ E, Giurgiu County, Romania). Fresh pitted fruits (with a mean value of moisture content of 76%) were dried in an oven (to a mean value of moisture content of 7%) and then ground using an electric grinder. The dried and ground fruits were stored in a dry place until extraction.

### 2.2. Chemicals

The extraction solvents were solutions consisting of analytical-grade ethanol, purchased from Merck KGaA (Darmstadt, Germany), and distilled water.

The reagents used in the Folin–Ciocalteu method, i.e., Folin–Ciocalteu reagent, sodium carbonate (Na_2_CO_3_), and gallic acid, and in the CUPRAC (cupric reducing antioxidant capacity) method, including copper (II) chloride (CuCl_2_), ammonia (NH_3_), acetic acid (CH_3_COOH), neocuproine, and Trolox, were provided by Merck KGaA.

The following 24 standard substances were used in the high-performance liquid chromatography (HPLC) analysis: caffeic acid (98%, HPLC grade, Merck KGaA), caftaric acid (Molekula GmbH, München, Germany), catechin hydrate (>98%, HPLC grade, Merck KGaA), chicoric acid (>98%, TCI, Tokyo, Japan), chlorogenic acid (primary reference standard, HWI Group, Ruelzheim, Germany), cyanidin chloride (>95%, HPLC grade, Merck KGaA), delphinidin chloride (analytical standard, Merck KGaA), ellagic acid dihydrate (>98%, HPLC grade, TCI), (−) epicatechin (>98%, HPLC grade, TCI), gallic acid (98%, Alfa Aesar, Haverhill, MA, USA), kaempferol (>97%, HPLC grade, Merck KGaA), malvidin chloride (>95%, HPLC grade, Merck KGaA), myricetin (>96%, HPLC grade), neochlorogenic acid (>98%, HPLC grade, TCI), pelargonidin chloride (Merck KGaA), protocatechuic acid (>98%, HPLC grade, TCI), quercetin (>95%, HPLC grade), rosmarinic acid (>98%, HPLC grade, Merck KGaA), rutin hydrate (95%, HPLC grade), syringic acid (>98.5%, Molekula GmbH), *trans*-ferulic acid (>98%, GC grade), *trans*-*p*-coumaric acid (analytical standard, Merck KGaA), *trans*-resveratrol (certified reference material, Merck KGaA), and vanillic acid (>98%, GC grade, TCI). The solvents used in the chromatographic analysis, including ethanol, acetonitrile, and formic acid, purchased from Merck KGaA, were used without additional purification.

### 2.3. Extraction Procedure

A Hielscher UP200H ultrasonic probe (200 W, 24 kHz) (Hielscher Ultrasonics, Teltow, Germany) was used for the extractions, and the temperature control was achieved using a Labo S200-H13 refrigerated/heating circulator (Labo, Istanbul, Türkiye). After extraction, the mixtures were centrifuged at 4000 rpm for 10 min in a Nahita 2698 digital centrifuge (Precisa, Sibiu, Romania), and the supernatants were kept at a temperature of 4 °C before analysis. Liquid (extraction solvent)/solid (dried plant material) ratio (*R_LS_*), ethanol concentration in the extraction solvent (*c_et_*), extraction temperature (*t*), pH of the extraction solvent (*pH*), amplitude of the ultrasonic device (*A*), and extraction time (*τ*) were chosen as quantitative factors of the extraction process.

### 2.4. Total Phenolic Content (TPC)

According to the experimental procedure described in our previous work [39], the *TPC* of the extracts was determined by the Folin–Ciocalteu method. The absorbance of a solution consisting of extract sample (0.25–0.5 mL), Folin–Ciocalteu reagent (1 mL), 20% Na_2_CO_3_ solution (1.5 mL), and distilled water (3 mL) was measured at 750 nm using a Jasco V 550 UV–Vis spectrophotometer (Jasco Corporation, Tokyo, Japan). The analysis of each extract was performed in triplicate, and the results were expressed as mg GAE (gallic acid equivalents)/g DM (dry matter).

### 2.5. Total Anthocyanin Content (TAC)

The *TAC* of the extracts was determined by a spectrophotometric method described in our previous work [39]. The absorbances of the extract solutions appropriately diluted with the extraction solvent were measured at 520 nm using the Jasco V 550 UV–Vis spectrophotometer. The analyses were performed in triplicate, and the results were expressed as mg C3GE (cyanidin-3-glucoside equivalents)/g DM.

### 2.6. Antioxidant Capacity (AC)

The *AC* was determined by the CUPRAC method [39]. The absorbance of a solution consisting of extract sample (*x* mL), CuCl_2_ (1 mL), ammonium acetate buffer solution (1 mL) obtained from NH_3_ and CH_3_COOH, neocuproine (1 mL), and distilled water (1.1 − *x* mL) was measured at 450 nm using the Jasco V 550 UV–Vis spectrophotometer. The analyses were performed in triplicate, and the results were expressed as mg TE (Trolox equivalents)/g DM.

### 2.7. Chemical Profile of Extracts

The chemical profile of blackthorn fruit extracts was evaluated based on HPLC analysis conducted using a Shimadzu Nexera-2 system (Shimadzu Corporation, Kyoto, Japan) equipped with a photodiode detector (SPD-M30A) with a Nucleosil C18 reversed-phase separation column. Details regarding HPLC analysis were given in our previous paper [39]. Table S1 contains relevant data of HPLC analysis, including standard substance retention times, quantification wavelength, calibration curves, and limits of detection and quantification. The analyses were performed in triplicate, and the results were expressed as mg/100 g DM.

### 2.8. Experimental Design, Statistical Analysis, and Optimization

PBD and CCD were selected as experimental designs. All measurements were conducted in triplicate, and the results were presented as either mean values or mean values ± standard deviations (*SD*). Statistical analysis and process factor optimization were performed using STATISTICA 10 software (Stat Soft Inc., Tulsa, OK, USA).

## 3. Results

### 3.1. Screening of Extraction Process Factors

**Figure 1:**
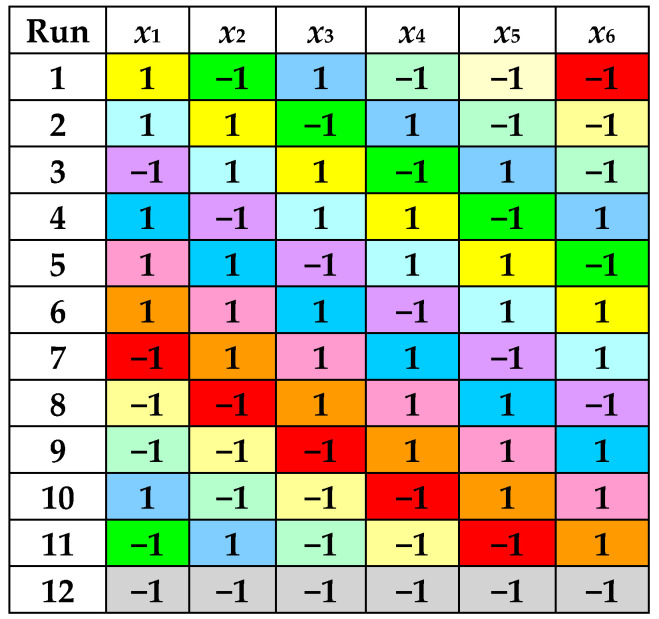
Levels of dimensionless factors in the PBD with 6 factors and 12 experimental runs.

According to a PBD with 6 factors, 12 experimental runs were performed at 2 levels of each process factor, i.e., *R_LS_* (5 cm^3^/g and 15 cm^3^/g), *c_et_* (30% and 70%), *t* (30 °C and 70 °C), *pH* (2 and 7), *A* (30% and 70%), and *τ* (5 min and 15 min). Levels of dimensionless factors defined by Equations (1)–(6) are specified in Figure 1 and Table 1.

(1) $$x_{1}=\frac{R_{LS}-10}{5}$$

(2) $$x_{2}=\frac{c_{et}-50}{20}$$

(3) $$x_{3}=\frac{t-50}{20}$$

(4) $$x_{4}=\frac{pH-4.5}{2.5}$$

(5) $$x_{5}=\frac{A-50}{20}$$

(6) $$x_{6}=\frac{\tau -10}{5}$$

Statistical models described by Equation (7) link the predicted response variables (*y_j_*_,*pr*_, *j* = 1…3), i.e., *y*_1,*pr*_ = *TPC_pr_*, *y*_2,*pr*_ = *TAC_pr_*, and *y*_3,*pr*_ = *AC_pr_*, to dimensionless process factors (*x_i_*, *i* = 1…6). Regression coefficients in Equation (7), i.e., *a_ij_* (*i* = 0…6, *j* = 1…3), were determined from mean experimental values (corresponding to triplicate measurements) of process response variables (*y_j_*_,*m*_, *j* = 1…3) specified in Table 1. The values of regression coefficients, coefficient of determination (*R_j_*^2^), adjusted coefficient of determination (*R_j,adj_*^2^), *F* statistic (*F_j_*), and *p_j_*-value for *F_j_*, which are also summarized in Table 1, indicate the following aspects:

(i) *y*_1,*pr*_ = *TPC_pr_* and *y*_3,*pr*_ = *AC_pr_* increase significantly with an increase in dimensionless liquid/solid ratio (*x*_1_) and extraction temperature (*x*_3_), decrease significantly with an increase in dimensionless ethanol concentration (*x*_2_), and do not vary significantly with the other 3 dimensionless factors; there is a very good agreement between experimental and predicted values of process response variables (*R_j_*^2^ ≥ 0.935, *R_j,adj_*^2^ ≥ 0.856, *F_j_* ≥ 11.91, and *p_j_* ≤ 0.008 for *j* = 1, 3);
(ii) *y*_2,*pr*_ = *TAC_pr_* decreases significantly with an increase in *x*_2_, whereas it does not vary significantly with the other 5 dimensionless factors; the statistical model defined by Equation (7) for *j* = 2 is statistically non-significant (*F* = 3.476 and *p* = 0.096).

(7) $$y_{j,pr}=a_{0j}+\sum_{i=1}^{6}a_{ij}x_{i},\;j=1\ldots 3$$

Consequently, the factors that significantly affected the extraction process were *R_LS_*, *c_et_*, and *t*. These factors were further optimized.

### 3.2. Optimization of Significant Process Factors

According to a CCD with 3 factors and 3 center points, 17 experimental runs were performed at 5 levels of process factors, i.e., *R_LS_* (4.95–15.1 cm^3^/g), *c_et_* (16.4–83.6%), and *t* (33.2–66.8 °C), as shown in Table 2. Values of other extraction parameters were as follows: *pH* = 7, *A* = 50%, and *τ* = 10 min. An extraction process performed at *pH* = 7 requires less chemical adjustment of the solvent by adding acids or bases, thus reducing production costs, technological process complexity, and environmental impact. Levels of dimensionless factors defined by Equations (8)–(10) and mean experimental values (corresponding to triplicate measurements) of process response variables (*Y_j_*_,*m*_, *j* = 1…3) are also specified in Table 2. Pearson correlation coefficients (*r*) indicated strong positive correlations between *Y*_1,*m*_ = *TPC_m_* and *Y*_3,*m*_ = *AC_m_* (*r* = 0.91), *Y*_1,*m*_ and *Y*_2,*m*_ = *TAC_m_* (*r* = 0.84), and *Y*_2,*m*_ and *Y*_3,*m*_ (*r* = 0.79).

(8) $$X_{1}=\frac{R_{LS}-10}{3}$$

(9) $$X_{2}=\frac{c_{et}-50}{20}$$

(10) $$X_{3}=\frac{t-50}{10}$$

Statistical models described by Equation (11) link the predicted response variables (*Y_j_*_,*pr*_, *j* = 1…3), i.e., *Y*_1,*pr*_ = *TPC_pr_*, *Y*_2,*pr*_ = *TAC_pr_*, and *Y*_3,*pr*_ = *AC_pr_*, to dimensionless process factors (*X_i_*, *i* = 1…3), *X_i_*^2^, and factor interactions (*X_i_X_k_*, *i*, *k* = 1…3, *k* > *i*). Regression coefficients in Equation (11), i.e., *b*_0*j*_, *b_ij_*, *b_iij_*, and *b_ikj_* (*i*, *k*, *j* = 1…3, *k* > *i*), were determined from experimental data (*Y_j_*_,*m*_, *j* = 1…3) specified in Table 2. The values of regression coefficients, *R_j_*^2^, *R_j,adj_*^2^, *F_j_*, and *p_j_*-value for *F_j_*, which are also summarized in Table 2, highlight the following:

(i) significant positive effects of dimensionless liquid/solid ratio (*X*_1_) and extraction temperature (*X*_3_) as well as a significant negative effect of dimensionless ethanol concentration (*X*_2_) on predicted response variables;
(ii) a significant negative effect of *X*_2_^2^ on *Y*_1,*pr*_ and *Y*_3,*pr*_;
(iii) a very good agreement between experimental and predicted values of process response variables (0.906 ≤ *R_j_*^2^ ≤ 0.965, 0.785 ≤ *R_j,adj_*^2^ ≤ 0.920, 7.498 ≤ *F_j_* ≤ 21.53, and 0.000 ≤ *p_j_* ≤ 0.007 for *j* = 1…3).

(11) $$Y_{j,pr}=b_{0j}+\sum_{i=1}^{3}b_{ij}X_{i}+\sum_{i=1}^{3}b_{iij}X_{i}^{2}+\sum_{\begin{array}{l} i,k=1\\ k>i \end{array}}^{3}b_{ikj}X_{i}X_{k},\;j=1\ldots 3$$

**Figure 2:**
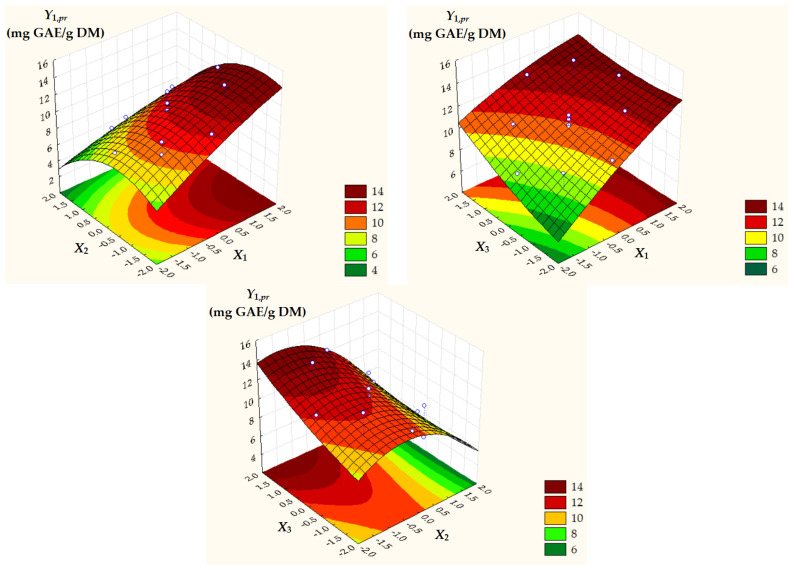
Surface and contour plots of *Y*_1,*pr*_ = *TPC_pr_* depending on dimensionless process factors; *X*_1_, liquid/solid ratio; *X*_2_, ethanol concentration; *X*_3_, extraction temperature.

**Figure 3:**
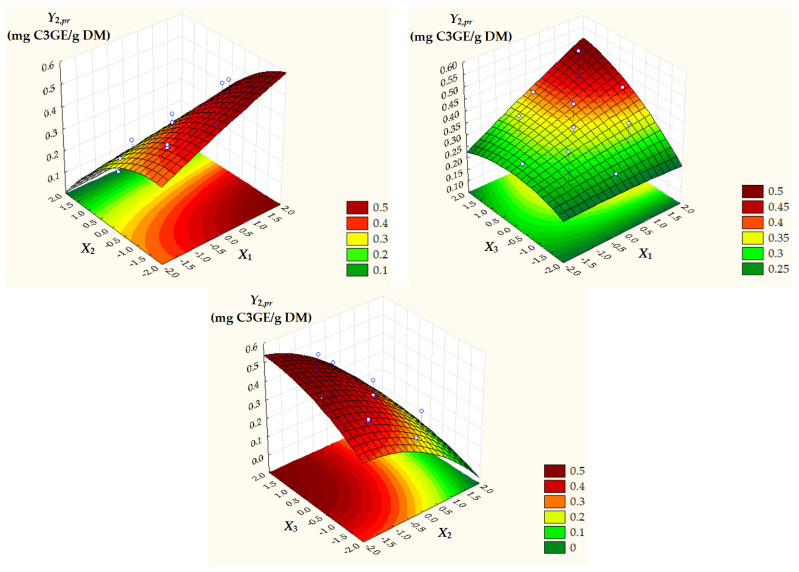
Surface and contour plots of *Y*_2,*pr*_ = *TAC_pr_* depending on dimensionless process factors; *X*_1_, liquid/solid ratio; *X*_2_, ethanol concentration; *X*_3_, extraction temperature.

**Figure 4:**
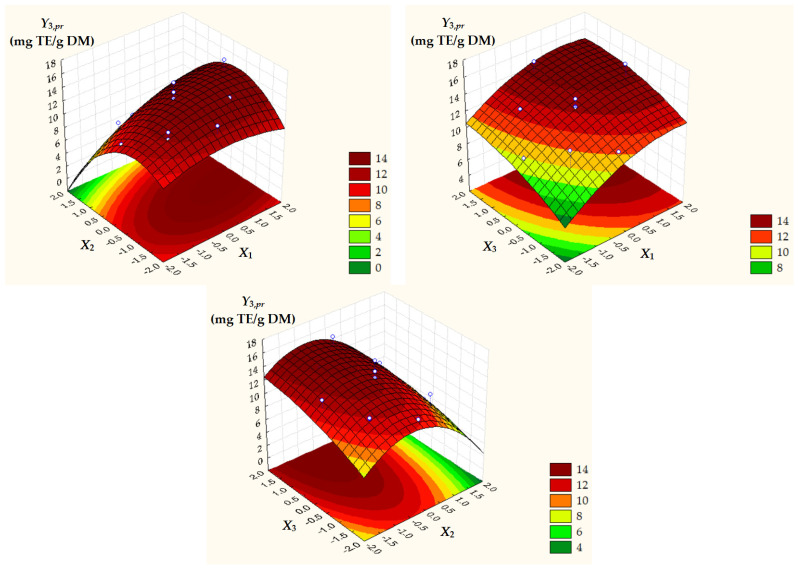
Surface and contour plots of *Y*_3,*pr*_ = *AC_pr_* depending on dimensionless process factors; *X*_1_, liquid/solid ratio; *X*_2_, ethanol concentration; *X*_3_, extraction temperature.

Surface and contour plots of process response variables depending on dimensionless process factors (Figure 2, Figure 3 and Figure 4) highlight an increase in all predicted responses with an increase in *X*_1_ and *X*_3_ as well as an increase in *Y*_2,*pr*_ = *TAC_pr_* with a decrease in *X*_2_.

**Figure 5:**
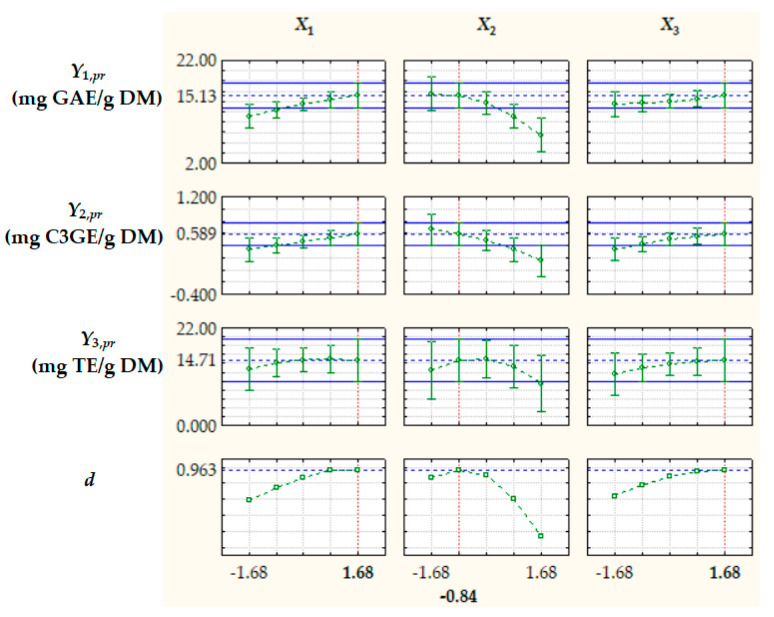
Predicted values of process response variables (*Y*_1,*pr*_ = *TPC_pr_*, *Y*_2,*pr*_ = *TAC_pr_*, and *Y*_3,*pr*_ = *AC_pr_*) and desirability function (*d*) at different levels of dimensionless process factors; *X*_1_, liquid/solid ratio; *X*_2_, ethanol concentration; *X*_3_, extraction temperature.

Optimization of extraction process factors, aiming at maximizing the process response variables, i.e., *TPC_pr_*, *TAC_pr_*, and *AC_pr_*, was based on the desirability function (*d*) approach [39]. The optimal levels of dimensionless factors were *X*_1,*opt*_ = 1.68, *X*_2,*opt*_ = −0.84, and *X*_3,*opt*_ = 1.68, corresponding to *R_LS_*_,*opt*_ = 15.1 cm^3^/g, *c_et_*_,*opt*_ = 33.2%, and *t_opt_* = 66.8 °C, and the value of *d* under optimal process conditions was 0.963 (Figure 5). The values of the process response variables predicted by Equation (11) at *X*_1,*opt*_, *X*_2,*opt*_, and *X*_3,*opt*_, i.e., *Y_j_*_,*pr*,*opt*_ (*j* = 1…3), are specified in Figure 5 and Table 3.

To validate the statistical models described by Equation (11), 3 extraction experimental runs were conducted at optimal levels of process factors. The mean values of experimental response variables at *R_LS_*_,*opt*_, *c_et_*_,*opt*_, and *t_opt_*, i.e., *Y_j_*_,*m*,*opt*_ (*j* = 1…3), related standard deviations (*SD_j_*), and values of percentage prediction error (*ε_j_*) defined by Equation (12) are summarized in Table 3. The values of percentage prediction error (−4.71% ≤ *ε_j_* ≤ 3.35%) and results of equal and unequal variance *t*-test (*p_j_* ≥ 0.09) indicated that *Y_j_*_,*m*,*opt*_ and *Y_j_*_,*pr*,*opt*_ were not significantly different, which proves the validity of statistical models defined by Equation (11).

(12) $$\varepsilon_{j}=100\frac{Y_{j,m,opt}-Y_{j,pr,opt}}{Y_{j,m,opt}},\;j=1\ldots 3$$

### 3.3. Chemical Profile of Extract Obtained Under Optimal Conditions

**Figure 6:**
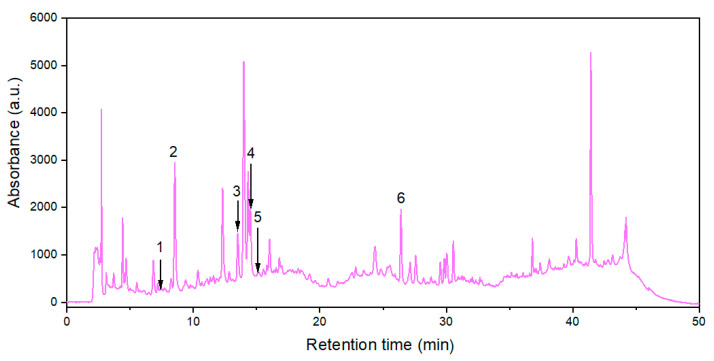
HPLC chromatogram (at 330 nm) for the extract obtained under optimal conditions: (1) protocatechuic acid; (2) neochlorogenic acid; (3) chlorogenic acid; (4) caffeic acid; (5) vanillic acid; (6) rutin.

Six polyphenols from 24 standard substances were identified in the extract obtained at optimal levels of extraction process factors, i.e., protocatechuic acid (PA), neochlorogenic acid (NCA), chlorogenic acid (CA), caffeic acid (CfA), vanillic acid (VA), and rutin (R). Their contents (expressed as mean values ± *SD*) in 3 extract samples prepared at *R_LS_*_,*opt*_ = 15.1 cm^3^/g, *c_et_*_,*opt*_ = 33.2%, and *t_opt_* = 66.8 °C are specified in Table 4, and related HPLC chromatogram is shown in Figure 6.

## 4. Discussion

Two experimental designs, i.e., PBD and CCD, were used to quantify the effects of process factors on extraction performance and to find the optimal levels of relevant factors. Six factors, including *R_LS_*, *c_et_*, *t*, *pH*, *A*, and *τ*, were screened based on a PBD. Only *R_LS_*, *c_et_*, and *t* had significant effects on extraction response variables, and the optimal levels of these factors were found using a CCD.

Depending on the experimental conditions used in PBD and CCD (*R_LS_* = 4.95–15.1 cm^3^/g, *c_et_* = 16.4–83.6%, *t* = 30–70 °C, *pH* = 2–7, *A* = 30–70%, and *τ* = 5–15 min), the following mean values of process response variables were found: *TPC_m_* = 6.279−14.89 mg GAE/g DM (1.620−3.842 mg GAE/g FM), *TAC_m_* = 0.093−0.557 mg C3GE/g DM (0.024−0.144 mg C3GE/g FM), and *AC_m_* = 5.343−16.75 mg TE/g DM (1.378−4.322 mg TE/g FM), where FM is fresh matter. These values and those of phenolic compound contents in the extract obtained under optimal conditions (Table 4) are consistent with data reported in the related literature [1,2,3,14,16,19,20,39,44,45,46]. The response variables of polyphenol extraction process can be significant affected by *R_LS_*, *t*, *τ*, number of extraction steps, and characteristics of blackthorn fruits (genotype, geographical origin, harvest time, pretreatment), extraction solvent (type, composition, pH), and extraction equipment. Values of *TPC*, *TAC*, and *AC* of blackthorn fruit extracts obtained by UAE in several studies are summarized in Table 5.

Opriș et al. (2021) [2] applied UAE (using a Transsonic T 310 ultrasonic probe, 95 W, 35 kHz) to obtain blackthorn fruit extracts (*R_LS_* = 10 cm^3^/g) from fruits supplied by SC Bio Boom Company SRL (Alexandria, Romania). According to a CCD with 3 process factors and 6 center points, 20 experimental runs were performed at 5 levels of each factor (*c_et_* = 23.2–56.8%, *t* = 33.2–66.8 °C, and *τ* = 1.6–18.4 min). The values of *TPC_m_*, i.e., 1.60–2.52 mg GAE/g DM, were significantly lower (up to 9 times) than those obtained in our study (6.772–14.45 mg GAE/g DM) under similar conditions used in CCD (*R_LS_* = 4.95–15.1 cm^3^/g, *c_et_* = 16.4–83.6%, *t* = 33.2–66.8 °C, and *τ* = 10 min), whereas the values of *AC_m_* obtained by DPPH assay (15.13–63.18 μmol TE/g DM) were quite similar to our results found using CUPRAC assay (6.584–15.67 mg TE/g DM = 26.24–62.46 μmol TE/g DM). Optimal extraction conditions (*c_et_*_,*opt*_ = 40%, *t_opt_* = 67 °C, and *τ_opt_* = 10 min) were quite close to those reported in the present study at *τ* = 10 min (*R_LS_*_,*opt*_ = 15.1 cm^3^/g, *c_et,opt_* = 33.2%, and *t_opt_* = 66.8 °C), whereas *TPC_m,opt_* (2.52 mg GAE/g DM) and *AC_m,opt_* (63.18 μmol TE/g DM) were lower than the mean values obtained in our study (14.45 mg GAE/g DM and 16.75 mg TE/g DM = 66.76 μmol TE/g DM).

Cosmulescu et al. (2017) [44] examined methanolic extracts of blackthorn fruits obtained in an ultrasonic bath (*R_LS_* = 1.67 cm^3^/g FM, *c_met_* = 70%, *t* = 25 °C, and *τ* = 60 min). Fruits collected from the Oltenia region (Romania) were frozen at −20 °C until extraction. The mean value of *TPC* (1.926 mg GAE/g FM) was within the range reported in our study (1.620–3.842 mg GAE/g FM), whereas that of *AC* obtained by DPPH assay (2.60 μmol TE/g FM) was significantly lower (2.1–6.6 times) than the mean values of *AC* found in our study using CUPRAC assay (1.378–4.322 mg TE/g FM = 5.492–17.23 μmol TE/g FM). Eleven phenolic acids (*p*-coumaric, ellagic, salycilic, sinapic, vanillic, chlorogenic, syringic, trans-cinnamic, ferulic, caffeic, and gallic) and five flavonoids (epicatechin, myricetin, quercetin, rutin, and catechin) were identified in methanolic extracts. The mean values of *VAC* (3.14 mg/100 g FM), *CAC* (2.29 mg/100 g FM), *CfAC* (0.44 mg/100 g FM), and *RC* (4.86 mg/100 g FM) were significantly higher than our findings (*VAC_m,opt_* = 0.95 mg/100 g FM, *CAC _m,opt_* = 0.50 mg/100 g FM, *CfAC_m,opt_* = 0.39 mg/100 g FM, and *RC_m,opt_* = 1.84 mg/100 g FM), probably due to a higher solubility of phenolic compounds in methanolic solutions and a longer extraction time.

González-de-Peredo et al. (2020) [1] applied UAE (using a Hielscher UP200S ultrasonic probe, 200 W, 24 kHz) to extract bioactive compounds from blackthorn fruits in aqueous solutions of methanol. The fruits harvested from Sesma (Navarre, Spain) were ground with a mixer, and the homogenous samples were stored in a freezer at −20 °C until use. According to a BBD with 6 process factors and 6 center points, 54 experimental runs were performed at 3 levels of each factor (*R_LS_* = 6.67–13.33 cm^3^/g FM, *c_met_* = 25–75%, *t* = 10–70 °C, *pH* = 2–7, *A* = 30–70%, and 0.2–0.7 s cycles). Experimental values of *TPC* (1.498–4.509 mg GAE/g FM) were similar to those found in our study (1.620–3.842 mg GAE/g FM). Optimal levels of process factors were as follows: *R_LS,opt_* = 10 cm^3^/g FM, *c_met,opt_* = 67%, *t_opt_* = 10 °C, *A_opt_* = 70%, *pH_opt_* = 7, and 0.2 s cycle. Four anthocyanins were identified in the extract samples, i.e., cyanidin 3-O-glucoside, cyanidin 3-O-rutinoside, peonidin 3-O-glucoside, and peonidin 3-O-rutinoside.

Dedić et al. (2021) [20] used UAE (performed in an ultrasonic homogenizer Iskra, UZ 4R) to obtain blackthorn fruit extracts (*R_LS_* = 10 cm^3^/g, *c_et_* = 100%, *t* = 30 °C, and *τ* = 20 min). The fruits were collected in April–September 2018 from 3 areas in Bosnia and Herzegovina (Borije, Trnovo, and Vareš), air-dried, and then ground into a fine powder. The mean values of *TPC* (2.766–4.116 mg GAE/g DM) were 1.5–5.4 times lower than those reported in our study, whereas the mean values of *TAC* (0.679–1.258 mg C3GE/g DM) were 1.2–13.5 times higher. The harvesting area had a significant influence on the mean values of *TPC*, *TAC*, and DPPH radical scavenging activity (39.15–71.56%).

Popović et al. (2020) [3] investigated the phenolic profile of 15 wild-growing blackthorn genotypes harvested in November 2015 from 3 regions in northern Serbia (Fruška Gora mountain slopes), i.e., 2 genotypes from Beška, 10 from Banstol, and 3 from an area close to Beška. Bioactive compounds were extracted from lyophilized fruits with ethanol solutions by sonication in an ultrasonic bath (*R_LS_* = 10 cm^3^/g, *c_et_* = 50%, *t* = 40 °C, and *τ* = 20 min). The mean values of *TPC* (11.10–30.43 mg GAE/g DM) of blackthorn extracts, which varied significantly depending on genotype, were similar to or higher than those obtained in our study (6.279–14.89 mg GAE/g DM). Three hydroxycinnamic acids, i.e., 3-caffeoylquinic (neochlorogenic) acid, 5-caffeoylquinic (chlorogenic) acid, and 3-p-coumaroylquinic acid, and eight flavonoids, i.e., four anthocyanins (cyanidin 3-glucoside, cyanidin 3-rutinoside, peonidin 3-glucoside, and peonidin 3-rutinoside), quercetin flavonol, and 3 quercetin derivatives, including quercetin-3-galactoside, quercetin-3-glucoside, and quercetin-3-rutinoside (rutin), were identified in the blackthorn fruit extracts. The most abundant classes of phenolic compounds were hydroxycinnamic acids and anthocyanins, especially neochlorogenic acid (*NCAC_m_* = 46.63–636.1 mg/100 g DM), cyanidine 3-glucoside (4.41–183.6 mg/100 g DM), and cyanidine 3-rutinoside (7.38–185.6 mg/100 g DM). The mean values of *NCAC* were significantly higher than the mean value obtained in our study at optimal levels of extraction process factors (*NCAC_m,opt_* = 4.88 mg/100 g DM), probably due to different genotypes, harvest periods, fruit drying methods, and extraction conditions. However, the mean values of *CAC* and *RC* found in our study at optimal levels of extraction process factors (*CAC_m,opt_* = 1.93 mg/100 g DM and *RC_m,opt_* = 7.12 mg/100 g DM) were in the ranges reported by Popović et al. (2020) [3] (*CAC_m_* = 1.70–30.92 mg/100 g DM and *RC_m_* = 2.96–32.85 mg/100 g DM).

Kotsou et al. (2023) [14] analyzed extracts of blackthorn fruits harvested in October 2023 from a hilly area near Spathades (Kalambaka, central Greece). Pitted fruits were cut into smaller pieces and then dried in a lyophilizer. Freeze-dried fruit slices were ground to a fine powder, and only particles with a mean diameter of 106 μm were used in the extraction experiments with aqueous ethanol solutions (*R_LS_* = 20 cm^3^/g). Twenty experimental runs were conducted at 5 levels of *c_et_* = 0–100%, *t* = 20–80 °C, and *τ* = 30–150 min, using the following techniques: (1) maceration under stirring at 500 rpm (ST), (2) pulsed electric field for 20 min (PEF) + ST, (3) ultrasound for 20 min (US) + ST, and (4) PEF + US + ST. Compared to our results, the values of *TPC* (3.08–24.20 mg GAE/g DM) were quite similar, those of *TAC* (0.012–0.149 mg C3GE/g DM) were similar or lower, and those of *NCAC* (169–457 mg/100 g DM) were significantly higher. Using the partial least squares (PLS) prediction model, the following optimal values of predicted response variables were determined by applying the desirability function approach: *TPC_opt,PLS_* = 31.5 mg GAE/g DM, *TAC_opt,PLS_* = 0.129 mg C3GE/g DM, and *NCAC_opt,PLS_* = 462 mg/100 g DM (at *c_et,opt,PLS_* = 25%, *t_opt,PLS_* = 35 °C, and *τ_opt,PLS_* = 80 min, using PEF + US + ST technique). The mean value of procatechuic acid content under optimal conditions determined by PLS (*PAC_m,opt,PLS_* = 3 mg/100 g DM) was significantly lower than that found in our study (*PAC_m,opt_* = 6.83 mg/100 g DM), whereas the mean values of chlorogenic acid content (*CAC_m,opt,PLS_* = 30 mg/100 g DM) and rutin content (*RC_m,opt,PLS_* = 19 mg/100 g DM) were significantly higher than our findings (*CAC_m,opt_* = 1.93 mg/100 g DM and *RC_m,opt_* = 7.12 mg/100 g DM).

Marčetić et al. (2022) [16] examined extracts from blackthorn fruits collected in September 2020 from two locations in Serbia, i.e., one in the western part (Ljig region) and the other in the center part (Crni Vrh mountain slope). The fruits were frozen at −20 °C and stored until maceration. After removing the stones, the fleshy parts of the fruits were chopped, rubbed in a mortar, and then extracted (*R_LS_* = 9 cm^3^/g FM) with methanol, ethanol (*c_et_* = 50%), or water under stirring on a shaker. The mean values of *TPC* for extracts prepared from fruits harvested from western and central Serbia (PSE1 and PSE2) were 3.214 and 2.170 mg GAE/g FM for methanolic extracts, 3.183 and 2.127 mg GAE/g FM for ethanolic extracts (in the range reported in our study, i.e., 1.620–3.842 mg GAE/g FM), and 1.646 and 1.522 mg GAE/g FM for aqueous extracts. The mean values of *TAC* for PSE1 and PSE2 were 0.095 and 0.144 mg C3GE/g FM for methanolic extracts, 0.041 and 0.079 mg C3GE/g FM for ethanolic extracts (in the range found in our study, i.e., 0.024–0.144 mg C3GE/g FM), and 0.022 and 0.007 mg C3GE/g FM for aqueous extracts. Statistical analysis indicated the following relevant aspects: (i) all values of *TPC_m,PSE_*_1_ were significantly higher (*p* < 0.05) than those of *TPC_m,PSE_*_2_; (ii) regardless of harvest region, the values of *TPC_m_* for methanolic and ethanolic extracts were similar (*p* ≥ 0.05) and significantly higher (up to 2 times) than those for aqueous extracts; (iii) in contrast to the findings for *TPC_m_*, for methanolic and ethanolic extracts, the values of *TAC_m,PSE_*_1_ were significantly lower (up to 2 times) than those of *TAC_m,PSE_*_2_; for aqueous extracts, the value of *TAC_m,PSE_*_1_ was significantly higher (3.3 times) than that of *TAC_m,PSE_*_2_; (iv) regardless of harvest region, the values of *TAC_m_* for methanolic extracts were significantly higher than those for the other extracts, and the values of *TAC_m_* for ethanolic extracts were significantly higher that those for aqueous extracts. Marčetić et al. (2022) [16] found 27 phenolic compounds in methanolic, ethanolic, and aqueous extracts prepared by maceration, including a hydroxybenzoic acid derivative (vanillic acid hexoside), hydroxycinnamic acid derivatives (caffeoylquinic acids, feruloylquinic acid, and caffeoylshikimic acid), and various flavonoids, e.g., anthocyanins (complexes of cyanidin and peonidin with a monosaccharide or disaccharide unit), glycosides of quercetin, methylquercetin, and kaempferol.

Drăghici-Popa et al. (2023) [39] prepared different extracts from blackthorn fruits harvested in October 2019 from Giurgiu County (Romania). Pitted fruits were pretreated with isooctane for 2 h at room temperature to remove the lipids, and then extracts were obtained using maceration under stirring as a conventional extraction method. Aqueous solutions of ethanol acidified with lactic acid were used as extraction solvents. The mean values of *TPC*, *TAC*, and *AC* at different levels of extraction factors (*R_LS_* = 10 cm^3^/g, *c_et_* = 50–100%, *t* = 30–82.5 °C, *τ* = 5–180 min, and 1100 rpm) were in the following ranges: *TPC_m_* = 4.70–37.23 mg GAE/g DM, *TAC_m_* = 0.034–0.415 mg C3GE/g DM, and *AC_m_* = 19.49–68.04 µmol TE/g DM = 4.890–17.07 mg TE/g DM. The results obtained in this study by applying UAE were within these ranges, except for *TAC_m_* (0.093–0.557 mg C3GE/g DM). The mean values of *PAC*, *VAC*, and *RC* obtained at *c_et_* = 50–100%, *t* = 30–60 °C, and *τ* = 30 min, i.e., *PAC_m_* = 2.08–4.47 mg/100 g DM, *VAC_m_* = 1.36–2.77 mg/100 g DM, and *RC_m_* = 1.87–3.77 mg/100 g DM, were lower than those found under optimal conditions in the present study (*PAC_m,opt_* = 6.83 mg/100 g DM, *VAC_m_* = 3.70 mg/100 g DM, and *RC_m_* = 7.12 mg/100 g DM), whereas *CfAC_m,opt_* = 1.51 mg/100 g DM was within the range reported in our previous paper (*CfAC_m_* = 1.05–3.36 mg/100 g DM) [39].

Dragović-Uzelac et al. (2007) [45] analyzed extracts of blackthorn fruits harvested from Croatia in 2005. Phenolic compounds were extracted in an aqueous solution of ethanol (*c_et_* = 80%) at *R_LS_* = 4 cm^3^/g FM. The mean value of *TPC* of extracts prepared from fruits harvested in November (0.859 mg GAE/g FM) was 57% higher than that for extracts obtained from fruits harvested in October (0.547 mg GAE/g FM) and 1.9–4.5 times lower than the values of *TPC_m_* found in our study (1.620–3.842 mg GAE/g FM).

Erturk et al. (2009) [46] studied three blackthorn genotypes harvested from Coruh Valley (Türkiye) in 2006 and 2007, i.e., dark purple (DPG), red (RG), and yellow (YG) skin color. They reported the following mean values of *TPC*: 4.07 mg GAE/g FM for DPG, 1.38 mg GAE/g FM for RG, and 1.17 mg GAE/g FM for YG. The mean value of *TPC* for DPG was similar to the maximum value of *TPC_m_* reported in our study (3.842 mg GAE/g FM), whereas the mean values of *TPC* for RG and YG were significantly lower than those obtained in our study.

Celik et al. (2017) [19] studied blackthorn fruit samples from the Van locality (Türkiye) harvested in June 2015 and stored at −20 °C until use. They reported the following phenolic compounds in blackthorn fruits: chlorogenic acid, caffeic acid, vanillic acid, gallic acid, syringic acid, *p*-coumaric acid, ferulic acid, protocatechuic acid, rutin, catechin, and phloridzin, the predominant phenolic compounds being chlorogenic acid and caffeic acid. The mean values of *CAC* (1.30 mg/100 g FM) and *CfAC* (1.07 mg/100 g FM) were about 3 times higher than those found in our study, whereas the mean values of *PAC* (0.026 mg/100 g FM), *VAC* (0.032 mg/100 g FM), and *RC* (0.047 mg/100 g FM) were 30–68 times lower.

Depending on the experimental conditions and characteristics of solid and liquid phases involved in the extraction process, various phenolic compounds were identified in the blackthorn fruit extracts, including [3,4,7,8,11,12,13,14,15,16,17,18,19,21,22,23,34,39,47]:

(i) hydroxycinnamic acids and derivatives, e.g., caffeic acid, *p*-coumaric acid, ferulic acid, 3-caffeoylquinic (neochlorogenic) acid, 5-caffeoylquinic (chlorogenic) acid, 3-*p*-coumaroylquinic acid, 3-feruloylquinic, and caffeoylshikimic acid;
(ii) hydroxybenzoic acids and derivatives, e.g., *p*-hydroxybenzoic acid, protocatechuic acid, gallic acid, syringic acid, vanillic acid, and vanillic acid hexoside;
(iii) flavonoids, e.g.,
  - anthocyanins (cyanidin, delphinidin, malvidin, pelargonidin, cyanidin 3-glucoside, cyanidin 3-acetylglucoside, cyanidin 3-rutinoside, cyanidin 3-pentoside, peonidin 3-glucoside, peonidin 3-acetylglucoside, peonidin 3-rutinoside, peonidin 3-pentoside);
  - flavonols (myricetin, kaempferol 3-glucoside, kaempferol 3-rutinoside, quercetin, quercetin 3-galactoside, quercetin 3-glucoside, quercetin 3-rutinoside (rutin), quercetin 3-xyloside, quercetin acetylrutinoside, quercetin hexoside, quercetin acetyhexoside, quercetin pentosylhexoside, quercetin rhamnosylhexoside, quercetin rhamnoside, quercetin hexosylrhamnoside);
  - flavanols (catechin, epicatechin, epigallocatechin, procyanidin B2);
  - flavanones (naringin);
  - flavones (apigenin, apigenin pentoside);
  - coumarins;
  - tannins.

## 5. Conclusions

Optimizing extraction factors is essential to achieve high process efficiency. This study aimed at optimizing the UAE process of phenolic compounds from Romanian blackthorn fruits using aqueous solutions of ethanol as green extraction solvents. Six quantitative factors (*R_LS_* = 5–15 cm^3^/g, *c_et_* = 30–70%, *t* = 30–70 °C, *pH* = 2–7, *A* = 30–70%, and *τ* = 5–15 min) were screened based on a PBD.

It was assessed that *R_LS_*, *c_et_*, and *t* significantly affected the process response variables (*TPC*, *TAC*, and *AC*), and these factors were then optimized by applying a CCD. Second-order polynomial models obtained based on experimental data (*TPC_m_* = 6.772–14.03 mg GAE/g DM, *TAC_m_* = 0.115–0.557 mg C3GE/g DM, and *AC_m_* = 6.584–15.67 mg TE/g DM for *R_LS_* = 4.95–15.1 cm^3^/g, *c_et_* = 16.4–83.6%, *t* = 33.2–66.8 °C, *pH* = 7, *A* = 50%, and *τ* = 10 min) highlighted significant positive effects of dimensionless *R_LS_* (*X*_1_) and *t* (*X*_3_) as well as a significant negative effect of dimensionless *c_et_* (*X*_2_) on predicted response variables (*TPC_pr_*, *TAC_pr_*, and *AC_pr_*). Moreover, *X*_2_^2^ had a significant negative effect on *TPC_pr_* and *AC_pr_*.

Under optimal conditions (*R_LS_*_,*opt*_ = 15.1 cm^3^/g, *c_et_*_,*opt*_ = 33.2%, and *t_opt_* = 66.8 °C), the experimental values (*TPC_m,opt_* = 14.45 mg GAE/g DM, *TAC_m,opt_* = 0.405 mg C3GE/g DM, and *AC_m,opt_* = 16.75 mg TE/g DM) and predicted values (*TPC_pr,opt_* = 15.13 mg GAE/g DM, *TAC_pr,opt_* = 0.589 mg C3GE/g DM, and *AC_pr,opt_* = 14.71 mg TE/g DM) of process response variables were in good agreement. Six polyphenols were identified by HPLC analysis in the extract obtained at optimal levels of process factors, including a flavonoid, i.e., rutin flavonol (7.12 ± 0.06 mg/100 g DM); two hydroxybenzoic acids, i.e., protocatechuic acid (6.83 ± 0.01 mg/100 g DM) and vanillic acid (3.70 ± 0.01 mg/100 g DM); and three hydroxycinnamic acids, i.e., neochlorogenic acid (4.88 ± 0.01 mg/100 g DM), chlorogenic acid (1.93 ± 0.02 mg/100 g DM), and caffeic acid (1.51 ± 0.01 mg/100 g DM).

The optimization procedure applied in this study can be used at other levels of extraction process factors or taking into account other factors. The extracts with enhanced concentrations of phenolic acids and flavonoids prepared under optimal operating conditions are valuable sources of antioxidants and could be used as ingredients in functional foods, dietary supplements, cosmetic and pharmaceutical products.

## Figures and Tables

**Table 1:** Mean experimental values of process response variables (*y_j_*_,*m*_, *j* = 1…3) and related values of regression coefficients (*a_ij_*, *i* = 0…6, *j* = 1…3), coefficient of determination (*R_j_*^2^), adjusted coefficient of determination (*R_j,adj_*^2^), *F* statistic (*F_j_*), and *p_j_*-value for *F_j_* at different levels of dimensionless process factors (*x_i_*, *i* = 1…6) in PBD.

Run	*x*_1_	*x*_2_	*x*_3_	*x*_4_	*x*_5_	*x*_6_	*y*_1,*m*_ = *TPC_m_* (mg GAE/g DM)	*y*_2,*m*_ = *TAC_m_* (mg C3GE/g DM)	*y*_3,*m*_ = *AC_m_* (mg TE/g DM)
1	1	−1	1	−1	−1	−1	13.30	0.436	11.52
2	1	1	−1	1	−1	−1	8.160	0.134	5.343
3	−1	1	1	−1	1	−1	7.058	0.260	8.001
4	1	−1	1	1	−1	1	14.89	0.450	13.35
5	1	1	−1	1	1	−1	10.23	0.186	8.036
6	1	1	1	−1	1	1	11.72	0.226	9.611
7	−1	1	1	1	−1	1	7.316	0.160	8.290
8	−1	−1	1	1	1	−1	9.157	0.279	9.937
9	−1	−1	−1	1	1	1	8.543	0.364	9.883
10	1	−1	−1	−1	1	1	13.21	0.301	10.83
11	−1	1	−1	−1	−1	1	6.279	0.093	6.077
12	−1	−1	−1	−1	−1	−1	8.074	0.185	7.338
*j*							1	2	3
*a*_0*j*_							**9.829**	**0.256**	**9.018**
*a*_1*j*_							**2.091**	0.033	**0.763**
*a*_2*j*_							**−1.367**	**−0.080**	**−1.458**
*a*_3*j*_							**0.744**	0.046	**1.099**
*a*_4*j*_							−0.112	0.006	0.122
*a*_5*j*_							0.159	0.013	0.366
*a*_6*j*_							0.498	0.010	0.656
*R_j_* ^2^							0.972	0.807	0.935
*R_j,adj_* ^2^							0.939	0.575	0.856
*F_j_*							29.13	3.476	11.91
*p_j_*							0.001	0.096	0.008

Statistically significant regression coefficients are highlighted in bold.

**Table 2:** Mean experimental values of process response variables (*Y_j_*_,*m*_, *j* = 1…3) and related values of regression coefficients (*b*_0*j*_, *b_ij_*, *b_iij_*, and *b_ikj_*, where *i*, *k*, *j* = 1…3, *k* > *i*), coefficient of determination (*R_j_*^2^), adjusted coefficient of determination (*R_j,adj_*^2^), *F* statistic (*F_j_*), and *p_j_*-value for *F_j_* at different levels of dimensionless process factors (*X_i_*, *i* = 1…3) in CCD.

Run	*R_LS_* (cm^3^/g)	*c_et_* (%)	*t* (°C)	*X*_1_	*X*_2_	*X*_3_	*Y*_1,*m*_ = *TPC_m_* (mg GAE/g DM)	*Y*_2,*m*_ = *TAC_m_* (mg C3GE/g DM)	*Y*_3,*m*_ = *AC_m_* (mg TE/g DM)
1	7	30	40	−1	−1	−1	9.191	0.377	11.49
2	7	30	60	−1	−1	1	10.61	0.390	12.54
3	7	70	40	−1	1	−1	6.818	0.115	7.288
4	7	70	60	−1	1	1	8.323	0.181	9.111
5	13	30	40	1	−1	−1	12.24	0.384	11.56
6	13	30	60	1	−1	1	14.03	0.557	13.67
7	13	70	40	1	1	−1	10.15	0.151	11.05
8	13	70	60	1	1	1	9.309	0.203	11.15
9	4.95	50	50	−1.68	0	0	8.492	0.298	9.708
10	15.1	50	50	1.68	0	0	13.25	0.432	15.67
11	10	16.4	50	0	−1.68	0	11.27	0.451	13.15
12	10	83.6	50	0	1.68	0	6.772	0.150	6.584
13	10	50	33.2	0	0	−1.68	10.12	0.276	11.05
14	10	50	66.8	0	0	1.68	12.95	0.393	15.42
15	10	50	50	0	0	0	10.76	0.328	13.07
16	10	50	50	0	0	0	10.55	0.355	12.82
17	10	50	50	0	0	0	11.62	0.355	14.08
*j*							1	2	3
*b*_0*j*_							**11.00**	**0.348**	**13.38**
*b*_1*j*_							**1.376**	**0.034**	**1.246**
*b*_11*j*_							−0.117	−0.002	−0.417
*b*_2*j*_							**−1.394**	**−0.115**	**−1.590**
*b*_22*j*_							**−0.771**	−0.025	**−1.415**
*b*_3*j*_							**0.631**	**0.037**	**0.910**
*b*_33*j*_							0.117	−0.012	−0.222
*b*_12*j*_							−0.268	−0.014	0.575
*b*_13*j*_							−0.248	0.018	−0.083
*b*_23*j*_							−0.318	−0.008	−0.155
*R_j_* ^2^							0.965	0.937	0.906
*R_j,adj_* ^2^							0.920	0.855	0.785
*F_j_*							21.53	11.51	7.498
*p_j_*							0.000	0.002	0.007

Statistically significant regression coefficients are highlighted in bold.

**Table 3:** Predicted and experimental values of extraction response variables under optimal process conditions.

*j*	Response Variable		Optimal Value		Percentage Prediction Error
	Symbol	Units	Predicted	Experimental	
			*Y_j,pr,opt_*	*Y_j,m,opt_* ± *SD_j_*	*ε_j_* (%)
1	*TPC*	mg GAE/g DM	15.13	14.45 ± 0.718	−4.71
2	*TAC*	mg C3GE/g DM	0.589	0.405 ± 0.057	−1.90
3	*AC*	mg TE/g DM	14.71	16.75 ± 1.144	3.35

**Table 4:** Polyphenol contents in the extract obtained under optimal conditions.

No.	Name	Symbol	Units	
			mg/100 g DM	mg/100 g FM
1	Protocatechuic acid content	*PAC_opt_*	6.83 ± 0.01	1.76 ± 0.00
2	Neochlorogenic acid content	*NCAC_opt_*	4.88 ± 0.01	1.26 ± 0.00
3	Chlorogenic acid content	*CAC_opt_*	1.93 ± 0.02	0.50 ± 0.01
4	Caffeic acid content	*CfAC_opt_*	1.51 ± 0.01	0.39 ± 0.00
5	Vanillic acid content	*VAC_opt_*	3.70 ± 0.01	0.95 ± 0.00
6	Rutin content	*RC_opt_*	7.12 ± 0.06	1.84 ± 0.02

DM, dry mater; FM, fresh matter.

**Table 5:** Comparison among *TPC*, *TAC*, and *AC* values reported in various studies.

Origin/Time of Harvest	Operation Conditions	*TPC*	*TAC*	*AC*	Reference
Romania/October 2023	Ultrasonic probe (200 W, 24 kHz); *R_LS_* = 4.95–15.1 cm^3^/g; *c_et_* = 16.4–83.6%; *t* = 30–70 °C; *pH* = 2–7; *A* = 30–70%; *τ* = 5–15 min	6.279–14.89 mg GAE/g DM 1.620–3.842 mg GAE/g FM	0.093–0.557 mg C3GE/g DM 0.024–0.144 mg C3GE/g FM	5.343–16.75 mg TE/g DM (a) (21.30–66.76 μmol TE/g DM) 1.378–4.322 mg TE/g FM (5.492–17.23 μmol TE/g FM)	This study
Romania	Ultrasonic probe (95 W, 35 kHz); *R_LS_* = 10 cm^3^/g; *c_et_* = 23.2–56.8%; *t* = 33.2–66.8 °C; *τ* = 1.6–18.4 min	1.60–2.52 mg GAE/g DM	-	15.13–63.18 μmol TE/g DM (b) (3.796–15.85 mg TE/g DM)	[2]
Romania/ autumn	Ultrasonic bath; *R_LS_* = 1.67 cm^3^/g FM; *c_met_* = 70%; *t* = 25 °C; *τ* = 60 min	1.926 ± 0.095 mg GAE/g FM	-	2.60 ± 0.10 μmol TE/g FM (b)	[44]
Spain	Ultrasonic probe (200 W, 24 kHz); *R_LS_* = 6.67–13.33 cm^3^/g FM; *c_met_* = 25–75%; *t* = 10–70 °C; *pH* = 2–7; *A* = 30–70%; 0.2–0.7 s cycles	1.498–4.509 mg GAE/g FM	0.093–0.266 mg CCE/g FM	-	[1]
Bosnia and Herzegovina/April– September 2018	Ultrasonic homogenizer; *R_LS_* = 10 cm^3^/g; *c_et_* = 100%; *t* = 30 °C; *τ* = 20 min	2.766–4.116 mg GAE/g DM	0.679–1.258 mg C3GE/g DM	39.15–71.56% (b)	[20]
Serbia/ November 2015	Ultrasonic bath; *R_LS_* = 10 cm^3^/g; *c_et_* = 50%; *t* = 40 °C; *τ* = 20 min	11.10–30.43 mg GAE/g DM	0.185–4.097 mg GAE/g DM	*IC*_50_ = 0.62–3.46 mg DM/mL (b) 7.06–25.27 mg AAE/g DM (c) (40.09–143.5 μmol AAE/g DM)	[3]
Greece/ October 2023	(1) maceration under stirring (ST); (2) pulsed electric field (PEF) + ST; (3) ultrasound (US) + ST; (4) PEF + US + ST; *R_LS_* = 20 cm^3^/g; *c_et_* = 0–100%; *t* = 20–80 °C; *τ* = 30–150 min	3.08–24.20 mg GAE/g DM	0.012–0.149 mg C3GE/g DM	200.15 ± 6.36 * μmol DPPH/g DM (b) 146.09 ± 3.20 * μmol AAE/g DM (c)	[14]

(a) CUPRAC (cupric reducing antioxidant capacity) assay; (b) DPPH (2,2-diphenyl-1-picrylhydrazyl) assay; (c) FRAP (ferric reducing antioxidant power) assay; AAE, ascorbic acid equivalents; CCE, cyanidine chloride equivalents; *A*, amplitude of the ultrasonic probe; *c_et_*/*c_met_*, ethanol/methanol concentration in the extraction solvent; *pH*, pH of the solvent; *R_LS_*, liquid/solid ratio; *t*, extraction temperature; *τ*, extraction time; * under optimal conditions (PEF + US + ST, *c_et,opt_* = 25%, *t_opt_* = 35 °C, and *τ_opt_* = 80 min).

## Data Availability

The original contributions presented in this study are included in the article and Supplementary Materials. Further inquiries can be directed to the corresponding author.

## Supplementary Materials

The following supporting information can be downloaded at: https://www.mdpi.com/article/10.3390/antiox14060680/s1, Table S1: Relevant data of HPLC analysis.
