# Supplementary material for: Optimization of Ultrasound-Assisted Extraction of Phenolic Compounds from Romanian Blackthorn (Prunus spinosa L.) Fruits
Source: Antioxidants (Basel). 2025 Jun 3;14(6):680. doi: 10.3390/antiox14060680 (PMC12189875; doi:10.3390/antiox14060680)
Supplement: Supplementary file 1 [file antioxidants-14-00680-s001.zip › antioxidants-3636746-supplementary.pdf]

antioxidants

Article

Optimization of Ultrasound-Assisted Extraction of Bioactive Compounds from Romanian Blackthorn (*Prunus Spinosa* L.) Fruits

Ana-Maria Drăghici-Popa[1], Oana Cristina Pârvulescu[2,*], Raluca Stan[1], and Ana-Maria Brezoiu[2]

[1] Department of Organic Chemistry, National University of Science and Technology POLITEHNICA Bucharest, 1-7 Gheorghe Polizu St., 011061 Bucharest, Romania; ana_maria.draghici@upb.ro (A.-M.D.-P.); raluca.stan@upb.ro (R.S.)
[2] Department of Chemical and Biochemical Engineering, National University of Science and Technology POLITEHNICA Bucharest, 1-7 Gheorghe Polizu St., 011061 Bucharest, Romania; anamaria.brezoiu@gmail.com (A.-M.B.)
* Correspondence: oana.parvulescu@yahoo.com (O.C.P.).

Supplementary material

Table S1. Relevant data of HPLC analysis.

No.	Compound	λ max (nm)	Retention time (min)	Calibration curve	R^2	LOD/LOQ (mg/L)	Linearity domain (mg/L)
1	Gallic acid	271	3.70	y=8371.14x-1207.10	0.9996	0.11/0.54	0.54-108.0
2	Protocatechuic acid	279	7.16	y=9007.30x-1186.03	0.9998	0.10/0.49	0.49-98.80
3	Neochlorogenic acid	326	8.67	y=7554.92x--1552.98	0.9993	0.50/1.00	1.00-100.0
4	Caftaric acid	326	10.92	y=3741.63x-1004.52	0.9990	0.10/0.50	0.50-100.0
5	Catechin hydrate	279	12.65	y=1704.55x-1484.97	0.9987	0.52/1.04	0.52-104.0
6	Chlorogenic acid	326	13.39	y=7634.01x-1686.39	0.9992	0.10/0.49	0.49-99.00
7	Vanillic acid	292	15.12	y=8457.44x-1216.69	0.9995	0.10/0.49	0.49-98.70
8	Caffeic acid	323	15.38	y=13188.1x-1501.42	0.9994	0.10/0.49	0.49-99.00
9	Syringic acid	271	16.80	y=6929.03x-667.27	0.9991	0.11/0.54	0.54-107.3
10	(-) Epicatechin	279	17.76	y=1585.49x-273.15	0.9993	0.47/0.94	0.94-93.50
11	Delphinidin chloride	529	21.21	y=13373.70x-5554.22	0.9984	0.11/0.59	0.59-118.0
12	*trans p*-coumaric acid	309	22.17	y=18220.60x-1642.10	0.9972	0.10/0.51	0.51-102.0
13	*Trans*-ferulic acid	323	25.46	y=13317.51x-3135.95	0.9997	0.10/0.52	0.52-104.0
14	Ellagic acid dihydrate	367	25.62	y=5128.27x-577.18	0.9995	0.10/0.51	0.51-71.12
15	Cyanidin chloride	524	25.67	y=13401.60x-2555.96	0.9990	0.08/0.42	0.42-84.00
16	Rutin hydrate	355	26.60	y=3813.02x-838.902	0.9992	0.49/0.99	0.99-99.80
17	Chicoric acid	330	29.18	y=10560.62x-1939.03	0.9996	0.10/0.51	0.51-101.6
18	Pelargonidin chloride	512	29.64	y=8921.84x-2511.85	0.9993	0.10/0.50	0.50-100.0
19	Malvidin chloride	535	30.90	y=7243.86x-2363.29	0.9983	0.09/0.46	0.46-92.00
20	Myricetin	373	31.87	y=9150.32x-1464.36	0.9995	0.10/0.48	0.48-95.00
21	Rosmarinic acid	330	32.36	y=7282.31x-633.293	0.9988	0.10/0.49	0.49-99.00
22	*trans*-Resveratrol	307	33.32	y=17601.10x-2585.25	0.9994	0.10/0.50	0.50-100.0
23	Quercetin	371	34.87	y=9898.83x-723.173	0.9997	0.10/0.49	0.49-99.00
24	Kaempferol	367	35.88	y=10549.70x-1296.00	0.9990	0.10/0.48	0.48-97.00

(LOD) limit of detection; (LOQ) limit of quantification.
